# Roles of Psychosocial Factors on the Association Between Online Social Networking Use Intensity and Depressive Symptoms Among Adolescents: Prospective Cohort Study

**DOI:** 10.2196/21316

**Published:** 2021-09-21

**Authors:** Ji-Bin Li, Li-Fen Feng, Anise M S Wu, Jin-Chen Mai, Yu-Xia Chen, Phoenix K H Mo, Joseph T F Lau

**Affiliations:** 1 Department of Clinical Research State Key Laboratory of Oncology in South China, Collaborative Innovation Center for Cancer Medicine Sun Yat-sen University Cancer Center Guangzhou China; 2 Center for Health Behaviours Research The Jockey Club School of Public Health and Primary Care The Chinese University of Hong Kong Hong Kong China; 3 Department of Statistics Government Affairs Service Center of Health Commission of Guangdong Province Guangzhou China; 4 Department of Psychology Faculty of Social Sciences University of Macau Macau China; 5 Department of Psychological Health Research Center for Health Promotion of Primary and Secondary School of Guangzhou Guangzhou China

**Keywords:** online social networking use intensity, depressive symptoms, psychosocial factors, mediation and suppression, longitudinal study

## Abstract

**Background:**

The potential mechanisms underlying the association between online social networking use intensity and depressive symptoms are unclear and underresearched.

**Objective:**

We aimed to investigate the potential roles of interpersonal psychosocial factors on the association between online social networking use intensity and depressive symptoms among early adolescents.

**Methods:**

A total of 4237 adolescents from a 9-month longitudinal study were included. Score changes (indicated as △) for the social function use intensity (SFUI) and entertainment function use intensity (EFUI) subscales of the Online Social Networking Activity Intensity Scale and for friendship quality, perceived family support, perceived friend support, parent–adolescent conflict, social nonconfidence, and depressive symptoms were analyzed. The potential mediation effects of unfavorable psychosocial factors and suppression effects of favorable psychosocial factors on the association of △SFUI with △CES-D and the association of △EFUI with △CES-D were tested using hierarchical regression models.

**Results:**

The association between △SFUI and △CES-D was partially mediated by △mother–adolescent conflict (mediation effect size 5.11%, *P*=.02) and △social nonconfidence (mediation effect size 20.97%, *P*<.001) but partially suppressed by △friendship quality, △perceived family support, and △perceived friend support, with suppression effects of –0.011 (*P*=.003), –0.009 (*P*=.003), and –0.022 (*P*<.001), respectively. The association between △EFUI and △CES-D was partially mediated by △social nonconfidence (mediation effect size 30.65%, *P*<.001) but partially suppressed by △perceived family support and △perceived friend support, with suppression effects of –0.036 (*P*<.001) and –0.039 (*P*<.001), respectively.

**Conclusions:**

The association between online social networking use intensity and depressive symptoms was partially mediated through the indirect increase in social nonconfidence and mother–adolescent conflict; however, better perceived social support and friendship quality would partially compensate for the harmful impact of online social networking use intensity on depressive symptoms among early adolescents.

## Introduction

Adolescent depression is a global challenge that is significantly associated with serious physical and psychological problems (eg, substance use, eating disorder, and suicide) [1-4]. A meta-analysis [5] estimated a pooled prevalence of depression of 24.3% among adolescents in mainland China (range 6.2%-64.8%); this prevalence was 24.6% at baseline and 26.6% 9 months later in our previous study [6] (which used the same data set as this paper). Various types of problematic internet use (eg, internet addiction and internet gaming disorders) aggravate adolescent depression [7-10]. The intensity of online social networking use also is positively associated with depressive symptoms among adolescents in several cross-sectional studies [11-13], and while it is necessary to have an understanding of the mechanisms underlying the association to address prevention and intervention, such an understanding is lacking. As cross-sectional studies are unable to establish temporal relationships, they cannot be used to investigate potential underlying mechanisms of the relationships between online social networking use intensity and depressive symptoms. Longitudinal studies are therefore warranted. Given that adolescents use online social networking mainly for social purposes (eg, communicating, seeking social support, and maintaining or establishing friendships) [14-16], we investigated whether favorable (friendship quality and social support) and unfavorable (conflict with parents and lack of social confidence) interpersonal attributes would mediate or suppress the association between online social networking use intensity measures and depressive symptoms in early adolescence, by using a prospective cohort study design [17-19].

Online social networking use can potentially lead to unfavorable and stressful interpersonal relationships. According to the displacement theory, internet use reduces adolescents’ communication with family members and social involvement, which then increases psychiatric symptoms [20]. Consistently, heavy online social networking use has been shown to reduce adolescents’ intimacy and time spent with their parents and families and to increase conflicts with parents [20,21]. For instance, college students who used online social networking to communicate with their parents reported higher levels of conflicts with their parents [22]. Parental control over adolescents’ online social networking use may also cause conflicts with adolescents [23]. Furthermore, heavy online social networking use was associated with fewer face-to-face interactions and social confidence among adolescents [24], possibly due to habitual deprivation of nonverbal cues (ie, voice tone, eye contact) and reduction in social presence, which may reduce self-efficacy in handling social interactions (social nonconfidence) [25]. As parent–adolescent conflict [26,27] and social nonconfidence [28] have also been positively associated with depressive symptoms, we contended that these 2 unfavorable interpersonal relationship factors would mediate the association between online social networking use intensity and depressive symptoms among adolescents.

Nevertheless, online social networking use may enhance friendship quality among adolescents; a study [23] showed that 43% of high school students perceived that online social networking use increased their closeness with friends; and another study [21] reported that 49% of the college students’ closest friends whom they met offline were also their closest friends in their online social networks. Online social networking use facilitates individuals’ social connectedness and self-disclosure [29,30]; it is a convenient and essential source of social support [14]. For instance, Facebook users reported better perceived social support than nonusers [31], and the length of time spent on Facebook and the number of Facebook friends were positively associated with perceived social support [32,33]; however, nonsignificant associations have also been reported [34,35]. The frequency of online social networking use has also been associated with higher perceived social support and enhanced friendship quality [31,36]. As friendship quality [37,38] and perceived social support [39,40] are protective against depressive symptoms, we contended that these 2 favorable interpersonal relationship factors would potentially suppress the positive association between online social networking use intensity and depressive symptoms among adolescents.

It has been reported that the patterns of social relationships and depressive symptoms are likely to differ between the younger and older adolescents [41]. As a key stage of early adolescence, the rapid physical (eg, early puberty), psychological (eg, concrete thinking but early moral concepts) and social (eg, emotional separation from parents, start of strong peer identification; early exploratory behaviors) developmental changes produce specific disease patterns, unusual presentations of symptoms, unique communication and management challenges, and they gradually establish identity and autonomy in the context of the social cultural environment [42,43]. Moreover, it has been observed that the risk of depression is low in childhood but increasing substantially with adolescence [44] and that meaningful risk begins in the early teens [45]. Therefore, from the perspective of early prevention, we chose young adolescents as the target population.

In this 9-month prospective cohort study, we aimed to test the significance of 3 potential mediators (ie, father–adolescent conflict, mother–adolescent conflict, and social nonconfidence) and 3 potential suppressors (ie, friendship quality, perceived family support, and perceived friend support) for the association between online social networking use intensity measures and depressive symptoms in early adolescence. Specifically, it was hypothesized that the associations between changes in 2 online social networking use intensity measures—social function use intensity (SFUI) and entertainment function use intensity (EFUI) and depressive symptoms would be partially mediated or suppressed by the aforementioned unfavorable or favorable interpersonal psychosocial factors. To the best of our knowledge, no studies have tested such mediation and suppression effects.

## Methods

### Participants and Procedures

This 9-month prospective cohort study was conducted in Guangzhou, South China. The methods have previously been described [6]. Briefly, 9 public secondary schools, 3 from each of the city’s 3 regions (ie, core, suburb, and outer suburb regions), were selected by convenience sampling, and all students of the seventh and eighth grade (ie, 7 and 8 years of formal education) were invited to participate in the study. Grade 9 students were not included into the study given that they were too busy preparing for the public examination for entrance to senior high schools and would leave their schools before the end of the 9-month follow-up period; it would thus be practically difficult to follow up such students. The anonymous structured questionnaire was self-administered in classroom settings without the presence of teachers. We announced that the return of the completed questionnaire implied consent to participate in the study; this information was also printed on the cover page of questionnaire. No incentive was given to the participants. Permission to conduct the survey was granted by the school principals. The Survey and Behavioral Research Ethics Committee of the Chinese University of Hong Kong approved the study.

A total of 5365 students (response rate 98.0%) completed the baseline questionnaire. The baseline and follow-up questionnaires completed by each student were matched based on the last 4 digits of the student’s home telephone number, the last 4 digits of the parents’ mobile phone number, the last 4 digits of the student’s identity card number, the student’s birthday, and the last letter of the student’s and parents’ name. A total of 4871 (follow-up rate 90.8%) of the 5365 students provided matched completed questionnaires at the follow-up survey. Students who were lost to follow-up were more likely to be females, be in their junior year of school, to not live with both parents, to have a perceived poor/very poor family financial situation, and to have lower self-reported academic performance (Multimedia Appendix 1). Students who did not use online social networking (n=643) were excluded from the data analysis. The effective sample size was 4237 in this study.

### Measurements

#### Background Variables

Participant sociodemographic information (ie, gender, grade, parental education levels, perceived family financial situation, and living arrangement with parents) and information about self-reported academic performance and perceived academic pressure were collected in the baseline survey.

#### Depression

Depressive symptoms were assessed using the Chinese version of the 20-item Center for Epidemiological Studies–Depression Scale (CES-D), which is an epidemiological screening tool, not a clinical diagnosis tool. All items were rated on a 4-point Likert scale from 0, representing none/rarely (less than 1 day), to 3, representing almost/all of the time (5-7 days), during the past week, with higher total scores (range 0 to 60) representing more depressive symptoms. Psychometric properties of the Chinese version of the CES-D have been demonstrated among Chinese adolescents [46]. The CES-D is commonly used in mental health studies [47], and depression defined by such scale is strongly associated with clinical diagnosis of depression [48]. In this study, the Cronbach α was 0.86 at baseline and 0.87 at follow-up, indicating good internal reliability in this study.

#### Online Social Networking Use Intensity

The intensity of online social networking use was measured using the Online Social Networking Activity Intensity Scale on a 5-point response scale ranging from 0 (never) to 4 (always). The scale was developed and validated among Chinese adolescents [49]. It measures SFUI (10 items; range: 0-40) and EFUI (4 items; range: 0-16). A higher score indicates a higher intensity of online social networking use. In this study, the Cronbach α for SFUI and EFUI were 0.88 and 0.60 at baseline and 0.89 and 0.62 at follow-up, which showed good internal reliability.

#### Friendship Quality

Friendship quality was measured using the 6-item peer-relationship subscale of the Children and Adolescent Quality of Life Scale, which has been widely used among Chinese adolescents [50,51]. Each item has 4 responses, and a higher score (range 6 to 24) indicates better friendship quality. In this study, the Cronbach α was 0.82 at baseline and 0.83 at follow-up, indicating good internal reliability.

#### Perceived Social Support

Perceived social support was measured using the Chinese version of the 12-item Multidimensional Scale of Perceived Social Support [52], including the dimensions of perceived family support (4 items) and perceived friends support (8 items). Items were rated on a 7-point Likert scale, from 1 (very strongly disagree) to 7 (very strongly agree). Higher scores indicated better perceived social support. In this study, Cronbach α values for both subscales were >.90 at both baseline and follow-up, indicating good internal reliability.

#### Parent–Adolescent Conflicts

The Chinese parent–adolescent conflict questionnaire includes 7 dimensions (ie, study, chores, expenses, living arrangement, appearance, relationship with other family members, and privacy) [53,54]. Frequency of conflict, from 1 (never) to 5 (almost every day), in the previous month, with the father and mother were rated separately. Higher scores (range 7-35) indicated more conflict with parents. In this study, Cronbach α values were 0.84 for father–adolescent conflict and 0.85 for mother–adolescent conflict at baseline and 0.86 and 0.87 at follow-up, indicating good internal reliability.

#### Social Nonconfidence

Social nonconfidence was measured using the 4-item social nonconfidence subscale of the Chinese version of the Social Skills Scale, which showed good psychometric properties among college students [28]. Social nonconfidence refers to a strong concern about what others think about oneself and the experience of nervous feelings during social occasions [55]. Items were ranked on a 5-point Likert scale, from 1 (definitely not like me) to 5 (exactly like me), with higher scores (range 4-20) indicating stronger social nonconfidence. In this study, the Cronbach α was 0.60 at baseline and 0.64 at follow-up, indicating acceptable internal reliability.

### Statistical Analysis

The associations between changes (indicated as △) in online social networking use intensity (SFUI and EFUI) and depressive symptoms were tested. Potential mediators of the associations included changes in father–adolescent conflict, mother–adolescent conflict, and social nonconfidence; potential suppressors included changes in friendship quality, perceived family support, and perceived friends support. Changes were determined by subtracting the scale scores at baseline from those at the 9-month follow-up.

Potential mediation or suppression effects were tested using hierarchical modeling based on the methods proposed by Baron and Kenny [56], which required the presence of significant associations between (1) △SFUI (or △EFUI) and △CES-D; (2) △SFUI (or △EFUI) and changes in potential mediators or suppressors; and (3) changes in potential mediators or suppressors and △CES-D after controlling for △SFUI (or △EFUI). The strength of the indirect effects (mediation or suppression) was estimated by the product *a × b*, where *a* is the regression coefficient that relates the independent variable to the potential mediator (or suppressors) and *b* is the regression coefficient that relates the mediators (or suppressors) to the dependent variable, with significance determined using Sobel *Z* [57,58]; *a* × *b* / (*a* × *b* + *c’*) reflects the magnitude of mediation effect size [57,59], where *c’* is the regression coefficients relating the mediators (or suppressors) to the dependent variable and the independent variable to the dependent variable in the same model. As the total effect of the suppressors could not be decomposed into indirect and direct proportions because it comprised associations in opposite directions, the proportion of the indirect effect could not be calculated; thus, only the suppressing effects (*a* × *b*) were reported. The interaction effects of gender for the associations between △SFUI (or △EFUI), changes in potential mediators (or suppressors) and △CES-D were further tested by including the interaction terms (gender multiplied by △SFUI, △EFUI, changes in potential mediators, or changes in potential suppressors).

Multilevel linear regression models (level 1: students, level 2: schools) with random intercepts were fitted to take into account the potential clustering effects from the schools. All models were adjusted for baseline background factors that were significantly associated with △CES-D in the univariate models (*P*<.05), and the unstandardized regression coefficients with standard errors are presented. Analyses were conducted using SAS software (version 9.4, SAS Institute). A 2-sided *P*<.05 was considered statistically significant.

## Results

### Sample Characteristics

In the baseline sample, 49.7% (2105/4237) of the participants were male, and 47.5% (2011/4237) were in the seventh grade. Approximately 48.3% (2047/4237) of students self-reported a good or very good family financial situation. The majority of students (88.4%, 3747/4237) lived with both parents. Approximately one-fifth (20.1%, 852/4237) reported lower academic performance, and 23.4% (993/4237) perceived heavy or very heavy academic pressure (Table 1).

**Table 1:** Background characteristics and their associations with change in Center for Epidemiological Studies–Depression Scale score by Univariate linear regression (n=4237).

Variables			n (%)		β (SE)		*P* value
**Sociodemographic variables**							
	**Gender**						
		Male	2105 (49.7)	—^a^			
		Female	2132 (50.3)	–0.12 (0.26)		.65	
	**Grade**						
		Seven	2011 (47.5)	—			
		Eight	2226 (52.5)	0.54 (0.26)		.04	
	**Father’s education level**						
		Primary school or below	273 (6.4)	—			
		Junior middle school	1425 (33.6)	0.53 (0.57)		.35	
		High middle school	1312 (31.0)	0.59 (0.58)		.31	
		University or above	1053 (24.9)	0.47 (0.60)		.43	
		Don't know	174 (4.1)	0.27 (0.84)		.74	
	**Mother’s education level**						
		Primary school or below	445 (10.5)	—			
		Junior middle school	1507 (35.6)	1.24 (0.47)		.008	
		High middle school	1199 (28.3)	1.16 (0.49)		.02	
		University or above	913 (21.5)	0.65 (0.51)		.20	
		Don't know	123 (4.1)	0.51 (0.77)		.51	
	**Family financial situation**						
		Very good/good	2047 (48.3)	—			
		Medium	2072 (48.9)	0.02 (0.27)		.93	
		Poor/very poor	118 (2.8)	0.27 (0.81)		.73	
	**Living with both parents**						
		Yes	3747 (88.4)	—			
		No	490 (11.6)	–0.71 (0.41)		.09	
**School-related variables**							
	**Academic performance**						
		Upper	1465 (34.6)	—			
		Medium	1920 (45.3)	–0.68 (0.30)		.02	
		Lower	852 (20.1)	–1.24 (0.37)		<.001	
	**Perceived academic pressure**						
		Nil/light	811 (19.1)	—			
		Average	2433 (57.5)	–0.36 (0.35)		.30	
		Heavy/very heavy	993 (23.4)	–1.46 (0.40)		<.001	

^a^Reference group.

During a 9-month follow-up period, the mean △CES-D was 0.48 (95% CI 0.22, 0.74; *P*<.001). There were slight significant reductions in the SFUI (*P*=.01) and EFUI scores (*P*<.001) and the scores of 3 potential suppressors (friendship quality: *P*=.001; perceived family support: *P*=.009; and perceived friend support: *P*<.001) and 2 potential mediators (father–adolescent conflict: *P*=.02; social nonconfidence: *P*<.001), whereas △mother–adolescent conflict from baseline to follow-up was not statistically significant (*P*=.21) (Table 2).

**Table 2:** Changes in Center for Epidemiological Studies–Depression, Social Function Use Intensity, Entertainment Function Use Intensity, and psychosocial factors scores from baseline to follow-up.

Variables			Baseline		Follow-up		Mean difference (95% CI)		*P* value for time		
Center for Epidemiological Studies–Depression score			15.1 (9.5)		15.6 (9.5)		0.48 (0.22, 0.74)		<.001		
**Online social networking use intensity**											
	Social Function Use Intensity	18.4 (8.3)		18.1 (8.4)		–0.31 (–0.55, –0.08)		.01			
	Entertainment Function Use Intensity	8.2 (3.1)		7.8 (3.2)		–0.46 (–0.56, –0.36)		<.001			
**Psychosocial factors**											
	Friendship quality	19.0 (3.4)		18.8 (3.4)		–0.17 (–0.27, –0.08)		.001			
	Perceived family support	20.4 (5.6)		20.2 (5.4)		–0.21 (–0.37, –0.05)		.009			
	Perceived friend support	42.0 (10.1)		40.7 (10.5)		–1.31 (–1.63, –1.00)		<.001			
	Father–adolescent conflict	12.9 (5.8)		12.7 (5.8)		–0.25 (–0.45, –0.05)		.02			
	Mother–adolescent conflict	14.5 (6.3)		14.6 (6.7)		0.15 (–0.08, 0.37)		.21			
	Social nonconfidence	12.8 (3.1)		12.6 (3.1)		–0.22 (–0.33, –0.12)		<.001			

### Associations Between △SFUI (or △EFUI) and △CES-D (or Changes in Psychosocial Factors)

After adjustment for grade, academic performance, and perceived academic pressure, we found that (1) both △SFUI and △EFUI were positively associated with △CES-D (Table 3); (2) all 3 favorable interpersonal variables (potential suppressors) were negatively associated with △CES-D, while all 3 unfavorable interpersonal variables (potential mediators) were positively associated with △CES-D (Table 4); (3) △SFUI was positively associated with all interpersonal variables, except △father–adolescent conflict (*P*=.12); and (4) △EFUI was positively associated with 2 favorable interpersonal variables (△perceived family support and △perceived friend support) and one unfavorable interpersonal variables (△social nonconfidence), but not with △friendship quality, △father–adolescent conflict, and △mother–adolescent conflict (Table 3).

Interaction effects of gender on (1) the association between △SFUI (or △EFUI) and △CES-D (*P*=.60; *P*=.41), (2) the association between △SFUI (or △EFUI) and change in psychosocial factors (*P*=.09-.94; *P*=.18-.93), and (3) the associations between psychosocial factors and △CES-D (*P* range: .30–.68) were nonsignificant, except for the interaction between gender and △SFUI on △father–adolescent conflict (interaction *P*=.006), between gender and △friendship quality on △CES-D (*P*=.03), and between gender and △perceived family support on △CES-D (interaction *P*=.04) (Multimedia Appendix 2 and Multimedia Appendix 3).

**Table 3:** Associations between △SFUI (or △EFUI) and △CES-D (or psychosocial factors) from multilevel linear regression models (n=4237).

Dependent variable		Independent variable									
		△Social Function Use Intensity					△Entertainment Function Use Intensity				
		Univariate β (SE)	*P* value	Adjusted^a^ β (SE)	*P* value	Univariate β (SE)		*P* value	Adjusted^a^ β (SE)	*P* value	
△CES-D^b^		0.08 (0.02)	<.001	0.07 (0.02)	<.001	0.14 (0.04)		<.001	0.14 (0.04)	<.001	
**Psychosocial factors**											
	△Friendship quality	0.02 (0.01)	.006	0.02 (0.01)	.004	0.02 (0.01)		.22	0.02 (0.02)	.06	
	△Perceived family support	0.03 (0.01)	.003	0.03 (0.01)	.002	0.15 (0.02)		<.001	0.15 (0.02)	<.001	
	△Perceived friend support	0.18 (0.02)	<.001	0.18 (0.02)	<.001	0.32 (0.05)		<.001	0.32 (0.05)	<.001	
	△Father–adolescent conflict	0.02 (0.01)	.08	0.02 (0.01)	.12	0.02 (0.03)		.56	0.01 (0.03)	.71	
	△Mother–adolescent conflict	0.04 (0.02)	.004	0.04 (0.02)	.006	0.06 (0.03)		.06	0.06 (0.03)	.10	
	△Social nonconfidence	0.04 (0.01)	<.001	0.04 (0.01)	<.001	0.12 (0.02)		<.001	0.12 (0.02)	<.001	

^a^Models were adjusted by grade, academic performance, and perceived study pressure.

^b^CES-D: Center for Epidemiological Studies–Depression scale.

**Table 4:** Associations between changes in psychosocial factors and △Center for Epidemiological Studies–Depression Scale (n=4237).

Psychosocial factors	Univariate		Adjusted^a^	
	β (SE)	*P* value	β (SE)	*P* value
△Friendship quality	–0.62 (0.04)	<.001	–0.61 (0.04)	<.001
△Perceived family support	–0.29 (0.02)	<.001	–0.29 (0.02)	<.001
△Perceived friend support	–0.11 (0.01)	<.001	–0.12 (0.01)	<.001
△Father–adolescent conflict	0.10 (0.02)	<.001	0.09 (0.02)	<.001
△Mother–adolescent conflict	0.10 (0.02)	<.001	0.09 (0.02)	<.001
△Social nonconfidence	0.38 (0.04)	<.001	0.37 (0.04)	<.001

^a^Models were adjusted by grade, academic performance, and perceived study pressure.

### Mediation and Suppression Effects Between △SFUI and △CES-D

When a potential mediator or suppressor was entered into the model that contained only △SFUI as the independent variable and had △CES-D as the dependent variable, a significant mediator resulted in a significant decrease in the regression coefficient, while a significant suppressor resulted in a significant increase in the regression coefficient between △SFUI and △CES-D. Accordingly, we found 2 significant mediators of the association between △SFUI and △CES-D: △mother–adolescent conflict (mediation effect size 5.11%, *P*=.02; model m1, Table 5) and △social nonconfidence (mediation effect size 20.97%, *P*<.001; model m2, Table 5). The combined mediation effect was approximately 25.33% (model m3).

In addition, we found that all 3 favorable interpersonal relationship variables were significant suppressors: △friendship quality (suppression effect: –0.011, *P*=.003; model s1, Table 6), △perceived family support (suppression effect: –0.009, *P*=.003; model s2), and △perceived friend support (suppression effect: –0.022, *P*<.001; model s3).

**Table 5:** Mediation effects of changes in negative psychosocial factors on the association between △SFUI (or △EFUI) and △Center for Epidemiological Studies–Depression Scale (n=4237) using multilevel linear regression models. All models were adjusted by grade, academic performance and perceived study pressure.

Model			β (SE)		*P* value		Mediation effect (the ratio of indirect effect to total effect)						
						Effect size (%)		Sobel *Z*		*P* value			
**Model m1**							5.11		2.43		.02		
	△SFUI^a^	0.07 (0.02)		<.001									
	△Mother–adolescent conflict	0.09 (0.02)		<.001									
**Model m2**							20.97		5.14		<.001		
	△SFUI	0.06 (0.02)		<.001									
	△Social nonconfidence	0.36 (0.04)		<.001									
**Model m3**							25.33		N/A^b^		N/A		
	△SFUI	0.06 (0.02)		<.001									
	△Mother–adolescent conflict	0.09 (0.02)		<.001									
	△Social nonconfidence	0.35 (0.04)		<.001									
**Model m4**							30.65		6.07		<.001		
	△EFUI^c^	0.10 (0.04)		<.001									
	△Social nonconfidence	0.36 (0.04)		<.001									

^a^SFUI: Social Function Use Intensity.

^b^N/A: not applicable.

^c^EFUI: Entertainment Function Use Intensity.

**Table 6:** Suppression effects of changes in positive psychosocial factors on the association between △SFUI (or △EFUI) and △Center for Epidemiological Studies–Depression Scale (n=4237) using multilevel linear regression models. All models were adjusted by grade, academic performance and perceived study pressure.

Model		β (SE)	*P* value	Suppression effect			
				Effect	Sobel *Z*	*P* value	
**Model s1**				–0.011	–2.95	.003	
	△SFUI^a^	0.08 (0.02)	<.001				
	△Friendship quality	–0.62 (0.04)	<.001				
**Model s2**				–0.009	–3.01	.003	
	△SFUI	0.08 (0.02)	<.001				
	△Perceived family support	–0.30 (0.02)	<.001				
**Model s3**				–0.022	–6.47	<.001	
	△SFUI	0.10 (0.02)	<.001				
	△Perceived friend support	–0.12 (0.01)	<.001				
**Model s4**				–0.036	–5.57	<.001	
	△EFUI^b^	0.18 (0.04)	<.001				
	△Perceived family support	–0.30 (0.02)	<.001				
**Model s5**				–0.039	–5.52	<.001	
	△EFUI	0.18 (0.04)	<.001				
	△Perceived friend support	–0.12 (0.01)	<.001				

^a^SFUI: Social Function Use Intensity.

^b^EFUI: Entertainment Function Use Intensity.

### Mediation and Suppression Effects Between △EFUI and △CES-D

△Social nonconfidence (mediation effect size: 30.65%, *P*<.001; model m4) was the only significant mediator for the association between △EFUI and △CES-D. Significant suppressors included △perceived family support (suppression effect –0.036, *P*<.001; model s4) and △perceived friend support (suppression effect –0.039, *P*<.001; model s5).

## Discussion

### Principal Findings

In this study, we found a significant increase in depressive symptoms (*P*<.001) among young adolescents during the 9-month period and positive associations between △SFUI (or △EFUI) and △CES-D. Moreover, associations were simultaneously partially mediated by some unfavorable psychosocial factors (ie, social nonconfidence and mother–adolescent conflict) and partially suppressed by some favorable psychosocial factors (ie, perceived social support and quality of friendship).

Consistent with our findings, previous studies conducted in the United States [60] and Iceland [61] had also reported a gradual increase in depressive symptoms among adolescents. The findings suggest that adolescents are vulnerable to gradual deterioration of their mental health and even chronic depression. Attention is required, given that depression may have lasting negative consequences for adolescents.

We found that, with increases in depressive symptoms, adolescents may have less favorable relationships (ie, reduced friendship quality and perceived social support) over time. Given that adolescents need to establish an autonomous self-identity, they may spend less time with their families, resulting in reduced perceived family support [62]. Our findings indicate that adolescents seem to face an additional challenge of having their relationships with their peers deteriorate over time, possibly due to the low quality of friendships formed online, the difference between online and general offline social connectedness [63], and important developmental changes during adolescence (eg, decrease in parental attachment, learning to form attachment relationships with peers, exploration of new environments) [64]. Fortunately, adolescents may gain social confidence over time, possibly because they have more practice in social interactions with other people. We observed that changes in interpersonal relationships were associated with changes in depressive symptoms. As social relationships are important determinants of mental, physical, and behavioral health among adolescents, longitudinal studies are warranted to understand other consequences of changes in such interpersonal relationships. In addition, we found a significant and slight decline in online social networking use intensity (both SFUI with *P*=.01 and EFUI with *P*<.001) over the 9-month period. To the best of our knowledge, our study is the first to document a longitudinal trend of online social networking use intensity among adolescents. We show that such changes were associated with changes in interpersonal relationships and depressive symptoms.

This study identified a number of important partial mediators and suppressors of the associations between △SFUI (or △EFUI) and △CES-D. First, △social nonconfidence partially mediated the associations of both △SFUI and △EFUI with △CES-D. This is a novel finding. An increase in online social networking use may thus increase, rather than reduce, social nonconfidence, which in turn increases depressive symptoms. While online social networking sites provide convenient platforms for establishing communication, contacts, and relationships among adolescents [65], they might simultaneously deprive adolescents of skills, time allocation, and experiences related to effective direct offline social interactions, resulting in social nonconfidence. Online social networking use does not involve the use of social and nonverbal cues; it may also weaken individuals’ desire for involvement in offline social activities and pleasure seeking [66]. Furthermore, social nonconfidence is potentially associated with isolation and other risk factors for depression, which might mediate the association between △SFUI (or △EFUI) and depression. Future research should distinguish between social nonconfidence in face-to-face versus online social interactions and reveal the relationship of social nonconfidence with online and offline social relationships.

Another important finding was that △father–adolescent conflict mediated the association of neither △SFUI nor △EFUI with △CES-D, while △mother–adolescent conflict partially mediated the association between △SFUI and △CES-D (a relatively small effect of 5.11%) but not between △EFUI and △CES-D. Thus, overall, conflict with parents is not a major mechanism explaining the associations between △SFUI (or △EFUI) and depressive symptoms. Furthermore, mother–adolescent conflict, but not father–adolescent conflict, played a role as a mediator. The difference between the 2 types of parental conflict was evident in the correlation results, which showed that △SFUI was positively associated with △mother–adolescent conflict, but not with △father–adolescent conflict, while △EFUI was not associated with either △father–adolescent conflict or △mother–adolescent conflict. We speculate that mothers tend to be more engaged in parenting than fathers; they also interact more frequently with adolescents concerning personal issues (eg, intensive online social networking use and poor time management) [67,68]. Such parenting may increase adolescent–mother conflict [69]. Additionally, heavy online social networking use may negatively impact adolescents’ academic performance [70,71] and result in adolescent conflicts with their mothers rather than with their fathers, given that mothers may be more involved and concerned about their children’s personal development (eg, academic performance) than fathers [72,73]. Thus, an increase in SFUI might result in adolescents’ more conflict with their mothers than with fathers.

△Friendship quality showed a suppression effect between △SFUI and △CES-D, but not between △EFUI and △CES-D, which is understandable, as SFUI reflects the social functions of online social networking use, while EFUI reflects the entertainment functions. Positive friendship is a protective factor against depressive symptoms among adolescents [74]. Online social networking is doubtlessly one of the most preferred platforms for communication among adolescents, through which they can be socially connected with peers regardless of time and place [75]. For instance, online social networking use can supplement offline interactions and communications with friends to improve friendship quality among adolescents. Mutual and interactive updating of information (eg, messages and photos) online increases perceived closeness with friends [76] and hence supports the maintenance of friendships, especially between friends that are geographically separated.

Equally important, both △perceived family support and △perceived friend support suppressed the association between changes in online social networking use intensity (both △SFUI and △EFUI) and △CES-D. Increases in SFUI and EFUI may enhance perceived support from family and friends, which in turn decreases the level of depressive symptoms. In this study, △SFUI and △EFUI were positively associated with perceived social support. Conceptually, online social networking use allows adolescents to express their needs for support readily by means of online self-disclosure (eg, communicating personal information, thoughts, and feelings with others online) [77,78], which has been shown to be correlated with perceived social support [29,79]. Simultaneously, online social networking use provides channels for others to provide social support to adolescents and for adolescents to receive such social support. Interestingly, △perceived family support, similar to △perceived friend support, was a suppressor. It is plausible that online social networking use is not limited to interaction with friends but also includes interaction with family members. The relationship of online social networking use with family support and its mechanisms require future research attention.

Importantly, △SFUI (or △EFUI) possessed both mediators (social nonconfidence and mother–adolescent conflict) that were risk factors for △CES-D and suppressors (friendship quality and perceived social support) that were protective factors against △CES-D. Stakeholders should be made aware that online social networking use by adolescents is neither good nor bad; related interventions should maximize protective suppressors and minimize harmful mediators of online social networking use among adolescents to transform harms into benefits. Attention should be paid to reducing harm that arises from social nonconfidence as a mediator, which was the strongest among all the mediators that were studied. Such interventions should provide training on social skills and communication skills. Conflict resolution strategies for adolescents and their mothers (eg, conciliation and positive problem-copying strategies) might also be useful [80]. In addition, given the high popularity of online social networking among adolescents, web-based platforms might be an innovative avenue to identify individuals at high risk for depression and can be a cost-effective tool to improve psychological well-being among adolescents [81].

### Limitations

This study has some limitations. Reporting bias (eg, social desirable bias and recall bias) might exist although most studies [21,22] used self-reported data on online social networking use. The survey may not be nationally representative, due to large geographic variations and its restriction to secondary school students. Our study focused on early adolescence (ie, secondary school students), caution needs to be used in generalizing the results to older adolescents. Future comparisons of this study’s findings with those of older adolescents are warranted. There were differences in some sociodemographic factors (ie, gender, grade, family financial situation, lives with both parents, and academic performance) between those who were followed up and lost to follow-up in our study. Although the influence of some of these factors (ie, grade, academic performance) have been considered in the multivariable analysis, interpretation of the findings should be made cautiously by considering the potential influence of these differences. Online and offline psychosocial relationships were not differentiated, whereas a study conducted in Hong Kong reported that perceived online social support failed to buffer stress for adolescents [82]. We investigated only perceived social support but no other forms of social support. The psychosocial factors represent self-perceptions, which were measured by widely used and validated scales. The measurements of parent–adolescent conflict could not identify whether the parent–adolescent conflict arose from online social networking use; further refinements in future studies were warranted. The relatively short follow-up time and involvement of only one follow-up (in the same school year) is a limitation; the interpretation of the findings should hence take the design into account (ie, that findings reflect short-term but not long term changes). We also did not include other types of interpersonal and personal mediators (or suppressors). The causal mediational sequence could not be derived from the 2-level study, and the follow-up period was relatively short. Moreover, the mediation and suppression effects found in this study were relatively small. Future multilevel research considering more comprehensive factors is warranted. Despite these limitations, our study has the strengths of being novel and using a longitudinal study design and well-validated instruments.

### Conclusion

Consistent with previous findings, an increase in online social networking use was associated with an increase in adolescents’ depressive symptoms. The risk effect was, however, partially a balance between the combination of mediation and suppression effects of psychosocial factors. The increase in online social networking use was associated with both unfavorable interpersonal situations (eg, increase in social nonconfidence) that increased depressive symptoms (mediators) and improvement in favorable interpersonal relationships (eg, perceived social support, friendship quality) that reduced depressive symptoms (suppressors). Future studies should clarify the relative contributions of the various interpersonal effects, and the findings of this study should be considered when designing interventions. Furthermore, we found that an increase in online social networking use also improved perceived family support. However, the mechanism, as well as the degree and role of the involvement of parents in adolescents’ online social networking use, have not been well studied in the literature; this involvement may be a potential moderator of the relationship between online social networking use and depressive symptoms, but the direction is unclear. Future studies are warranted. Finally, our findings provide a reminder for various stakeholders to eliminate the stigma against adolescents who are heavy online social networking users, given that online social networking use can generate both harmful and protective effects against depressive symptoms. The understanding or perception that online social networking use is associated with depressive symptoms needs to be reviewed and elaborated.

## Appendix

Multimedia Appendix 1 Attrition analysis for participants lost to follow-up.

Multimedia Appendix 2 Interaction effects of gender for the associations between △SFUI (or △EFUI) and △CES-D (or changes in psychosocial factors).

Multimedia Appendix 3 Interaction effects of gender for the associations between changes in psychosocial factors and △CES-D.

## Abbreviations

CES-D: Center for Epidemiological Studies–Depression Scale
EFUI: Entertainment Function Use Intensity
SFUI: Social Function Use Intensity
