# Supplementary material for: Roles of Psychosocial Factors on the Association Between Online Social Networking Use Intensity and Depressive Symptoms Among Adolescents: Prospective Cohort Study
Source: J Med Internet Res. 2021 Sep 21;23(9):e21316. doi: 10.2196/21316 (PMC8493459; doi:10.2196/21316)
Supplement: Multimedia Appendix 1 [file jmir_v23i9e21316_app1.docx]

**Multimedia appendix 1. Supplementary table.**

Table S1. Attrition analysis for participants lost to follow-up

|  | | Followed up (*n*=4871) | Lost to follow-up (*n*=494) | *P ^a^* |
| --- | --- | --- | --- | --- |
| **Socio-demographic variables** | |  |  |  |
| **Gender** | |  |  |  |
|  | Male | 2352 (48.3) | 181 (36.6) | <.001 |
|  | Female | 2519 (51.7) | 313 (63.4) |  |
| **Grade** | |  |  |  |
|  | Seven | 2331 (47.9) | 261 (52.8) | .04 |
|  | Eight | 2540 (52.1) | 233 (47.2) |  |
| **Father’s education level** | |  |  |  |
|  | Primary school or below | 326 (6.7) | 30 (6.1) | .98 |
|  | Junior secondary school | 1644 (33.8) | 172 (34.8) |  |
|  | Senior secondary school | 1495 (30.7) | 151 (30.6) |  |
|  | College or above | 1196 (24.5) | 121 (24.5) |  |
|  | Don't know | 210 (4.3) | 20 (4.0) |  |
| **Mother’s education level** | |  |  |  |
|  | Primary school or below | 532 (10.9) | 56 (11.3) | .95 |
|  | Junior secondary school | 1740 (35.7) | 169 (34.2) |  |
|  | Senior secondary school | 1360 (27.9) | 137 (27.7) |  |
|  | College or above | 1032 (21.2) | 111 (22.5) |  |
|  | Don't know | 207 (4.3) | 21 (4.3) |  |
| **Family financial situation** | |  |  |  |
|  | Very good/Good | 2308 (47.4) | 211 (42.7) | .005 |
|  | Average | 2409 (49.4) | 255 (51.6) |  |
|  | Poor/Very poor | 154 (3.2) | 28 (5.7) |  |
| **Lives with both parents** | |  |  |  |
|  | Yes | 4300 (88.3) | 412 (83.4) | .002 |
|  | No | 571 (11.7) | 82 (16.6) |  |
| **School-related variables** | |  |  |  |
| **Academic performance** | |  |  |  |
|  | Upper | 1667 (34.2) | 150 (30.4) | <.001 |
|  | Medium | 2198 (45.1) | 198 (40.1) |  |
|  | Lower | 1006 (20.7) | 146 (29.5) |  |
| **Perceived academic pressure** | |  |  |  |
|  | Nil/light | 949 (19.5) | 85 (17.2) | .15 |
|  | General | 2777 (57.0) | 275 (55.7) |  |
|  | Heavy/Very heavy | 1145 (23.5) | 134 (27.1) |  |

*^a^* *P* values were obtained by chi-square test.
