# Supplementary material for: Roles of Psychosocial Factors on the Association Between Online Social Networking Use Intensity and Depressive Symptoms Among Adolescents: Prospective Cohort Study
Source: J Med Internet Res. 2021 Sep 21;23(9):e21316. doi: 10.2196/21316 (PMC8493459; doi:10.2196/21316)
Supplement: Multimedia Appendix 2 [file jmir_v23i9e21316_app2.docx]

**Multimedia appendix 2. Supplementary table.**

Table S2. Interaction effects of gender for the associations between △SFUI/△EFUI and **△**CES-D/change in psychosocial factors

| Dependent variables | *P* for interaction terms *^a^* | |
| --- | --- | --- |
|  | gender×**△**SFUI | gender×**△**EFUI |
| **△**CES-D | 0.6 | 0.41 |
| **△**Friendship quality | 0.34 | 0.87 |
| **△**Perceived family support | 0.31 | 0.18 |
| **△**Perceived friend support | 0.94 | 0.36 |
| **△**Father-adolescent conflict | 0.006 | 0.24 |
| **△**Mother-adolescent conflict | 0.09 | 0.56 |
| **△**Social non-confidence | 0.26 | 0.93 |

CES-D: Center for Epidemiological Studies-Depression scale.

*^a^* all models were adjusted of grade, academic performance and perceived study pressure
