# Supplementary material for: Roles of Psychosocial Factors on the Association Between Online Social Networking Use Intensity and Depressive Symptoms Among Adolescents: Prospective Cohort Study
Source: J Med Internet Res. 2021 Sep 21;23(9):e21316. doi: 10.2196/21316 (PMC8493459; doi:10.2196/21316)
Supplement: Multimedia Appendix 3 [file jmir_v23i9e21316_app3.docx]

**Multimedia appendix 3. Supplementary table.**

Table S3. Interaction effects of gender for the associations between changes in psychosocial factors and **△**CES-D

| Interaction terms | DV=**△**CES-D |
| --- | --- |
|  | *P* for interaction terms *^a^* |
| Gender×**△**friendship quality | 0.03 |
| Gender×**△**perceived family support | 0.04 |
| Gender×**△**perceived friend support | 0.30 |
| Gender×**△**father-adolescent conflict | 0.46 |
| Gender×**△**mother-adolescent conflict | 0.40 |
| Gender×**△**social non-confidence | 0.68 |

CES-D: Center for Epidemiological Studies-Depression scale.

*^a^* all models were adjusted of grade, academic performance and perceived study pressure
